# Moon Jellyfish Mucin and Collagen Attenuate Catabolic Activity in Chondrocytes but Show Limited Efficacy in an Osteoarthritis Rat Model

**DOI:** 10.3390/ijms262210920

**Published:** 2025-11-11

**Authors:** Haruka Omura, Eriko Toyoda, Takayuki Baba, Ryoka Uchiyama, Masahiko Watanabe, Masato Sato

**Affiliations:** 1 Department of Orthopaedic Surgery, Surgical Science, Tokai University School of Medicine, 143 Shimokasuya, Isehara 259-1193, Kanagawa, Japan; hrk.omr@gmail.com (H.O.);; 2 Center for Musculoskeletal Innovative Research and Advancement (C-MiRA), Tokai University Graduate School, 143 Shimokasuya, Isehara 259-1193, Kanagawa, Japan; 3 Jelly Labo, Inc., 1-17-37, Oizumigakuen-cho, Nerima, Tokyo 178-0061, Japan; 4 The Institute of Medical Sciences, 143 Shimokasuya, Isehara 259-1193, Kanagawa, Japan

**Keywords:** jellyfish mucin, jellyfish collagen, osteoarthritis of the knee

## Abstract

Cartilage regeneration has long been a major challenge in the treatment of osteoarthritis (OA). Aiming to develop a simple outpatient treatment for knee OA, we have demonstrated the potential of combining Nomura’s jellyfish mucin (JM) and hyaluronic acid (HA) to contribute to cartilage repair and regeneration in chondrocytes. In this study, we examined the effects of moon jellyfish JM and jellyfish collagen (JC) on chondrocytes. Polydactyly-derived chondrocytes (PDs), obtained from polydactyly surgery, were used. PDs were cultured in media supplemented with JM or JC, harvested, and evaluated by RT-qPCR. The effects of simultaneous addition of the inflammatory cytokine IL-1β were also examined. Furthermore, the effects on rat articular cartilage were investigated. A mono-iodoacetate (MIA) model was created by intra-articular injection in 6-week-old rats, followed by four intra-articular injections. Evaluations were performed using macroscopic observation and histological assessment with the OARSI scoring system. In vitro, the addition of JM or JC significantly affected the expression of *ACAN*, *MMP3*, and *ADAMTS5*. However, in vivo, intra-articular injection of JM alone did not significantly suppress cartilage degeneration in MIA-induced OA model rats. Both JM and JC may contribute to the suppression of cartilage degeneration as well as to cartilage repair and regeneration, even in the absence of HA. However, further studies are needed to clarify the optimal conditions, such as dosage, timing, and delivery method, that are required to achieve these effects in articular cartilage.

## 1. Introduction

Osteoarthritis (OA) is one of the most common joint diseases. In many regions, the number of patients has been dramatically increasing, with a prevalence reaching 20–30% of the adult population [1]. Besides aging, factors such as obesity [2] and trauma [3,4] are also known to contribute to the onset of OA, and it may develop even in younger individuals. Although novel genes potentially involved in OA have been identified, the full pathogenesis of OA remains unclear [5]. In OA, proliferating synovial tissue produces inflammatory cytokines such as interleukins (ILs), which further promote arthritis progression and cause structural changes including cartilage degeneration. Therefore, these cytokines are currently considered key factors in OA pathogenesis [6]. Since such structural changes are irreversible, regeneration of cartilage tissue has long been a major challenge in medicine.

As mentioned above, in OA, inflammatory cytokines secreted from proliferating synovium induce catabolic effects on articular cartilage, thereby driving the progression of arthritis [7]. Thus, suppression of the action of inflammatory cytokines is considered a potential therapeutic strategy for OA [8,9].

One point-of-care option for OA is intra-articular injection of sodium hyaluronate (HA). Numerous reports have demonstrated the usefulness of HA in reducing pain, improving quality of life, and protecting the articular cartilage surface [10,11,12]. Other local injection therapies that have attracted attention as novel and effective treatments include intra-articular injection of platelet-rich plasma (PRP), purified from autologous blood [13], and intra-articular injection of adipose-derived stem cells (ASC) harvested from autologous subcutaneous tissue [14]. Our institution has also been investigating the effects of PRP in both basic and clinical studies. Wasai et al. reported comparative findings on PRP based on cytokine concentrations and clinical outcomes such as KOOS [15].

JM, owing to its structural similarity to glycoproteins present on the surface of articular cartilage, is expected to be beneficial for articular cartilage [16,17]. Its use as a scaffold for chondrocytes and soft tissue engraftment has also been investigated [18,19]. Similarly, JC has been suggested to be useful as a scaffold for wound healing and cell proliferation [20,21]. Our institution has previously examined and reported the effects of JM derived from Nomura’s jellyfish (Nemopilema nomurai) [16]. Although mucins are widely found in nature in animals such as rats, sheep, and cattle [22], no large-scale production of animal-derived mucins has been established. Nomura’s jellyfish was previously designated as a pest following sudden mass appearances, which significantly impacted the fishing industry and led to mandatory capture. Research into JM began as one of the attempts to explore effective utilization of the captured Nomura’s jellyfish. However, the population of Nomura’s jellyfish has currently decreased. Therefore, research is continuing using Moon jellyfish, which is now more readily available. Moon jellyfish can be stably collected without difficulty in individual harvesting and are considered appropriate as a medical resource [23].

In this study, we investigated whether moon jellyfish–derived JM, like that derived from Nomura’s jellyfish, is also beneficial for articular cartilage. The effects of JC were similarly examined. Polydactyly-derived chondrocytes (PDs) were cultured for 24 h under conditions with JM or JC, harvested, and analyzed by quantitative reverse transcription polymerase chain reaction (RT-qPCR) to assess changes in gene expression. Based on these results, we further examined the effects of JM on rat knee articular cartilage. A monosodium iodoacetate (MIA) injection was administered into the knee joints of 6-week-old rats to create an MIA model (OAK model) [24]. Subsequently, intra-articular injections of each agent were administered four times, and the effects were histologically evaluated.

## 2. Results

### 2.1. Measurement of Endotoxin Concentration

Because JM and JC are substances of animal origin and endotoxin concentration is an important factor in regenerative medicine, their respective endotoxin concentrations were measured. The endotoxin concentration of JM measured using the Limulus ES-II single test Wako in our hospital was 17.47 EU/mL, and the endotoxin concentration of JC was 4.84 EU/mL.

### 2.2. Effects of JM and JC on Cell Proliferation

**Figure 1:**
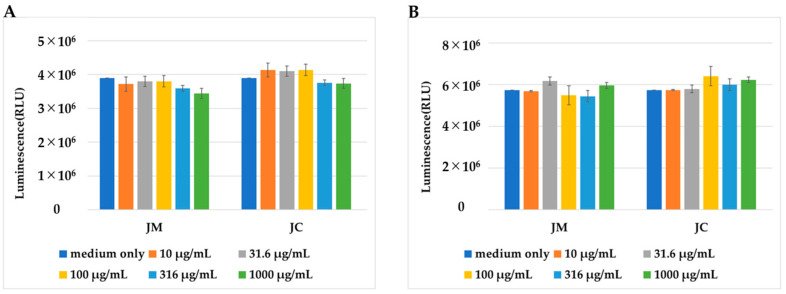
Comparison of the number of PDs cultured in medium containing JM or JC at the concentrations indicated. (**A**) Number of cells 24 h after changing to the medium containing JM or JC. (**B**) Number of cells 48 h after changing to the medium containing JM or JC.

We first examined the effects of JM and JC on the proliferation of PDs. PDs were thawed and seeded with PD medium, and 24 h later, the medium was replaced with a medium in which JM and JC were added at various concentrations. Cell proliferation was measured using the CellTiter-Glo 2.0 Assay (Promega, Madison, WI, USA) at 24 h and 48 h after replacement of the medium. Cell proliferation did not differ significantly for PDs grown in PD medium alone or with JM or JC added to the medium (Figure 1). These findings led us to conclude that JM and JC had no cell-growth-inhibitory effect.

### 2.3. Effects of JM on Gene Expression in PDs

**Figure 2:**
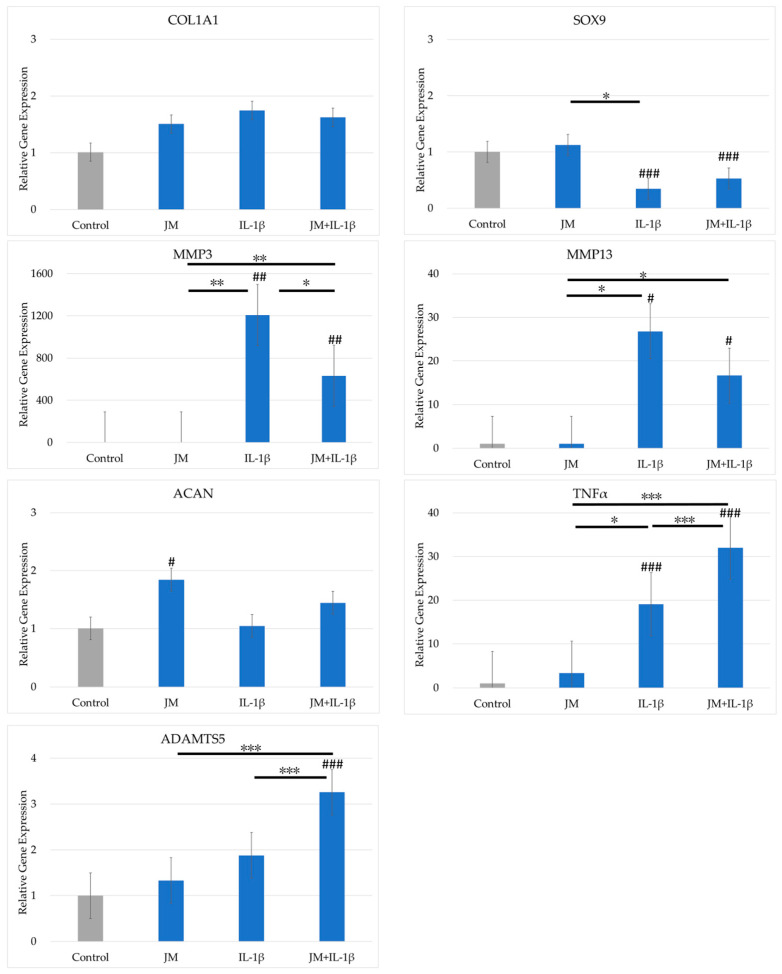
Comparison of gene expression in polydactyly-derived cells cultured with JM with and without interleukin 1β (IL-1β). The vertical axis shows the relative gene expression quantified using the 2^−ΔΔCT^ method. Gene expression was normalized by GAPDH expression. # indicates a significant difference compared with the control (### *p* < 0.005, ## *p* < 0.01, # *p* < 0.05). The horizontal bar and * indicate a difference between the groups (*** *p* < 0.005, ** *p* < 0.01, * *p* < 0.05).

The effects of JM on gene expression in cultured PDs are shown in Figure 2. The expression levels of *COL2A1* and *TGFβ* were below the detection limit. *COL1A1* expression did not differ significantly between the four conditions. *SOX9* expression was significantly reduced by addition of IL-1β to the culture medium, but did not differ between cultures with and without JM. The expression levels of *MMP3*, *MMP13*, and *TNFα* increased significantly when IL-1β was added to the culture. By contrast, *MMP3* expression was significantly lower when JM and IL-1β were added together compared with IL-1β added alone. *TNFα* expression was significantly higher in cultures with IL-1β alone and further higher in cultures with JM and IL-1β added at the same time. The expression of *ACAN* increased significantly with addition of JM. *ADAMTS5* and *TNFα* expression was significantly higher when IL-1β and JM were added simultaneously compared with IL-1β alone.

### 2.4. Effects of JC on Gene Expression in PDs

**Figure 3:**
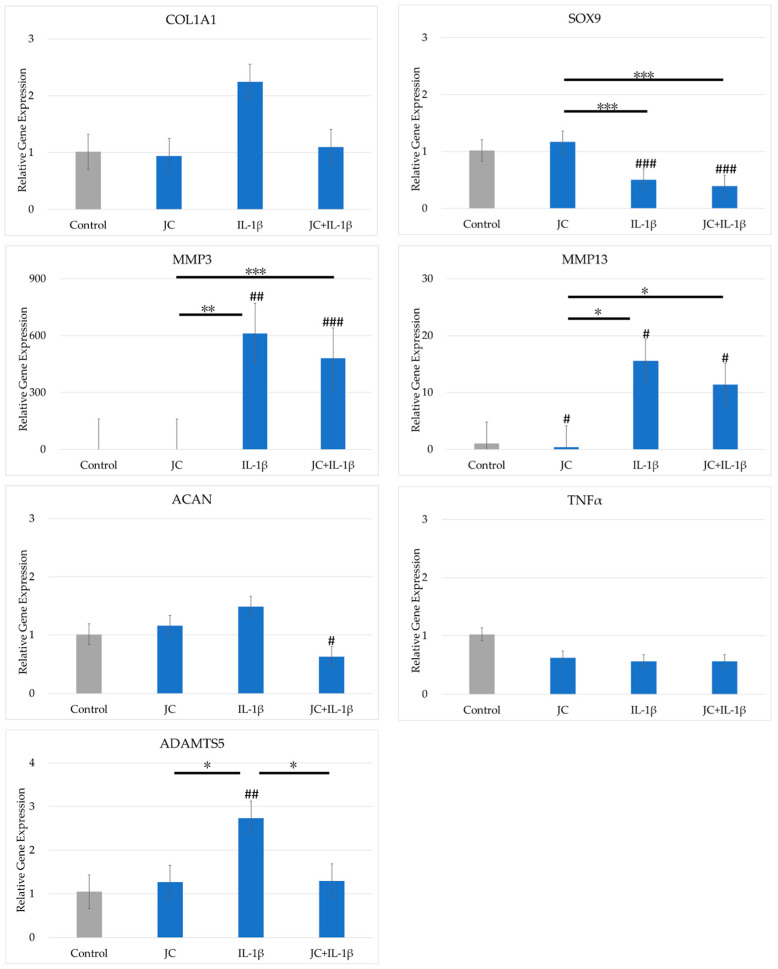
Comparison of gene expression in polydactyly-derived cells cultured with JC with and without interleukin 1β (IL-1β). The vertical axis shows the relative gene expression quantified using the 2^−ΔΔCT^ method. Gene expression was normalized by GAPDH expression. # indicates a significant difference compared with the control (### *p* < 0.005, ## *p* < 0.01, # *p* < 0.05). The horizontal bar and * indicate a difference between the groups (*** *p* < 0.005, ** *p* < 0.01, * *p* < 0.05).

The effects of JC on gene expression in cultured PDs are shown in Figure 3. The expression levels of *COL2A1* and *TGFβ* were below the detection limit. The expression levels of *COL1A1*, *COL2A1*, and *TNFα* did not differ significantly between the four conditions. *SOX9* expression was reduced by addition of IL-1β to the culture medium but did not differ between cultures with and without JC. The expression levels of *MMP3*, *MMP13*, and *ADAMTS5* increased significantly with the addition of IL-1β. Among these genes, only *ADAMTS5* expression was lower for the combination of IL-1β and JC added together than for IL-1β alone.

### 2.5. Effects of the Combination of JM and JC on Gene Expression in PDs

**Figure 4:**
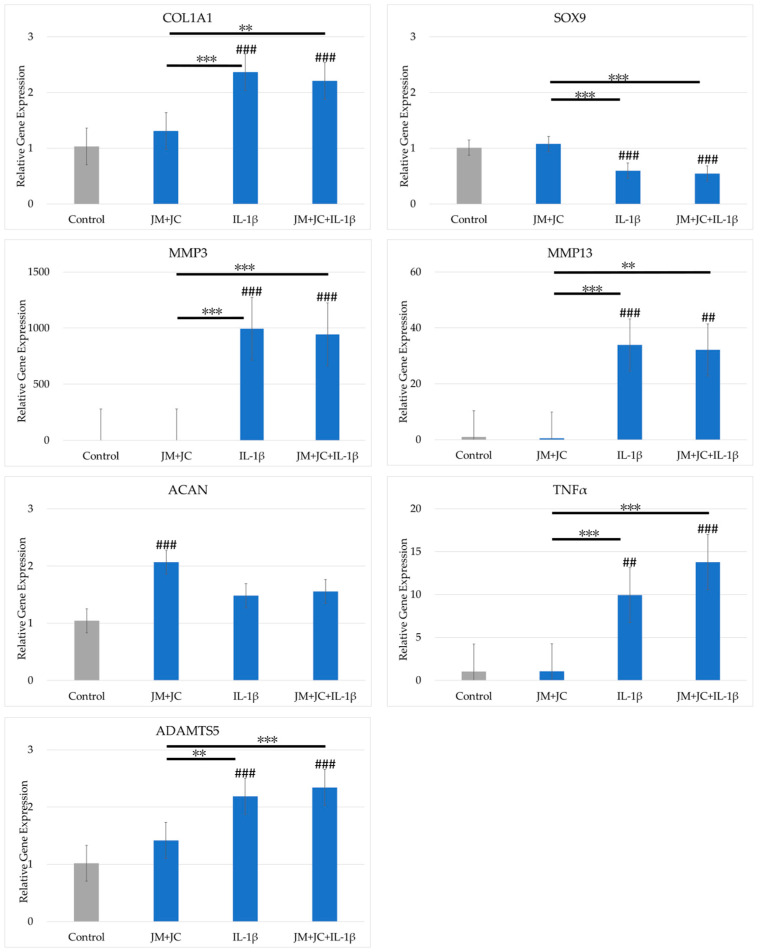
Comparison of gene expression in polydactyly-derived cells cultured with JM and JC together with and without interleukin 1β (IL-1β). The vertical axis shows the relative gene expression quantified using the 2^−ΔΔCT^ method. Gene expression was normalized by GAPDH expression. # indicates a significant difference compared with the control (### *p* < 0.005, ## *p* < 0.01). The horizontal bar and * indicate a difference between the groups (*** *p* < 0.005, ** *p* < 0.01).

The results of the combination of JM and JC on gene expression in cultured PDs are shown in Figure 4. *COL2A1* and *TGFβ* expression levels were below the detection limit. The expression levels of *COL1A1*, *MMP3*, *MMP13*, *TNFα*, and *ADAMTS5* were significantly increased by the addition of IL-1β, but did not differ between cultures with and without JM and JC together. *SOX9* expression was significantly reduced by IL-1β, but did not differ between cultures with and without JM and JC added together. *ACAN* expression increased significantly when JM and JC were added together, but did not differ significantly for the other three conditions.

### 2.6. Changes in Weight-Bearing Ratio Following Intra-Articular Injections in MIA Model Rats

**Figure 5:**
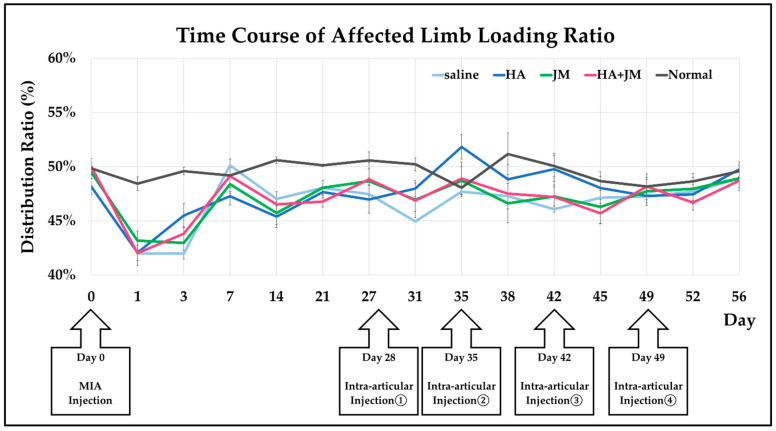
Timing of intra-articular injections and changes in weight-bearing ratio of the affected limb.

The day of MIA injection for establishing the MIA model (OAK model) rats [24] was defined as Day 0. The timing of each intra-articular injection and the changes in weight-bearing ratio of the affected limb in each group are shown in Figure 5. In the four groups that received MIA injection, the weight-bearing ratio of the affected limb decreased compared with the normal group that did not receive MIA injection, and this tendency persisted until Day 31. However, after Day 35, no clear differences were observed among the five groups.

### 2.7. Comparison of Macroscopic Scores of Articular Cartilage

**Figure 6:**
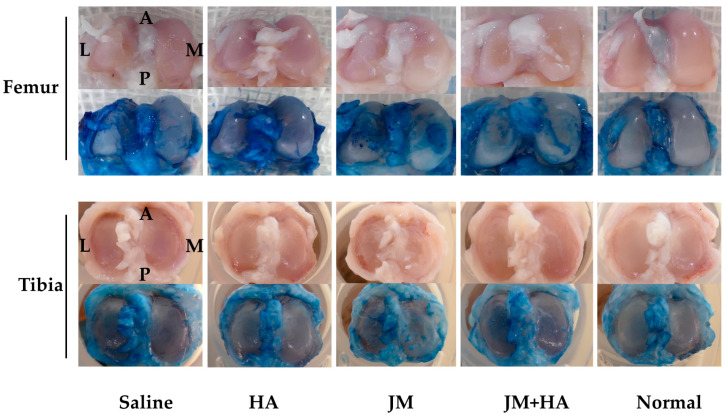

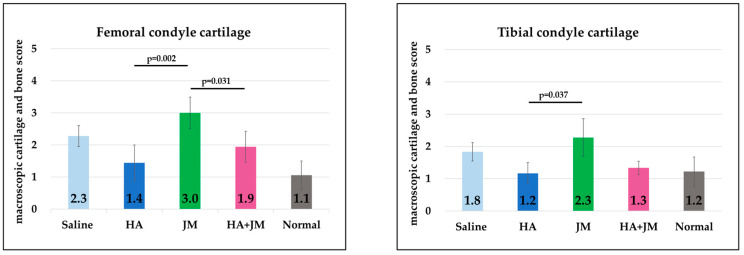
Results of macroscopic evaluation. In the photos above, the upper panel shows unstained samples, and the lower panel shows samples stained with indigo carmine. The abbreviations in the figure indicate the anatomical directions of the joint: A for Anterior, P for Posterior, M for Medial, and L for Lateral. The graph below shows the results of scoring using the macroscopic cartilage and bone score.

On Day 56, rats were sacrificed, and the articular cartilage surfaces were evaluated macroscopically. Both the femoral and tibial sides were assessed using the macroscopic cartilage and bone score [24] (Figure 6). Significant differences were observed between the HA group and the JM-containing groups on both the femur and tibia. On the femoral side, a significant difference was also observed between the JM group and the HA + JM group.

### 2.8. Comparison of Histological Scores of Articular Cartilage

**Figure 7:**
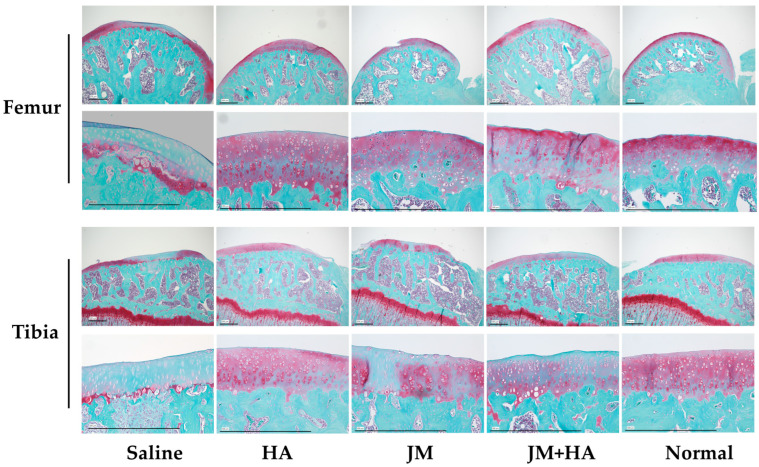

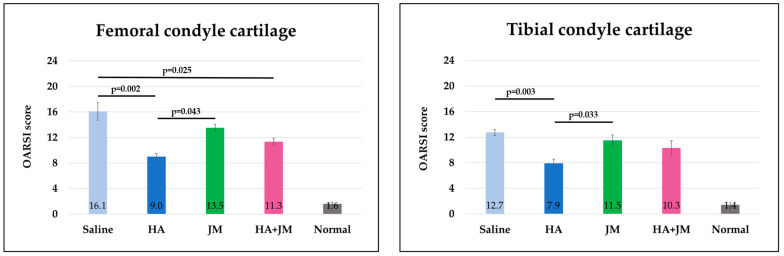
Photographs of safranin-stained tissue (low and high magnification) are shown. The scale bar in the lower-left corner of each image represents 500 µm. The graph below shows the results of histological evaluation using the OARSI score.

Both the femoral and tibial sides were evaluated using the OARSI score [25] (Figure 7). Significant differences were observed between the saline group and the HA group, and between the HA and JM groups, on both the femur and tibia. On the femoral side, a significant difference was also observed between the saline group and the HA + JM group.

## 3. Discussion

JM shares structural features with lubricin, a glycoprotein with a mucin domain that protects articular cartilage by forming a lubricating membrane and reducing surface friction [16,26]. Both molecules contain tandem repeat regions of 7–8 amino acids and short oligosaccharide side chains. Lubricin is also recognized as an early biomarker of joint injury [27]. JM further resembles human mucin 5AC [28], but because it lacks sialic acid and consists almost entirely of mucin sequences, it is unlikely to elicit adverse immune reactions such as allergies [16]. Human mucins exert diverse physiological roles, including ocular surface protection and facilitation of trace element absorption [29,30,31]. These structural and functional similarities provide a rationale for investigating JM as a potential chondroprotective agent, although few studies have directly evaluated its effects on articular cartilage.

Collagen has also been linked to OA treatment. Oral supplementation with collagen can improve joint pain [32], and ingested collagen accumulates in cartilage [33]. However, direct intra-articular administration raises safety concerns: antibodies binding strongly to collagen may trigger pain [34], and seafood-derived collagen can remain allergenic even after heat treatment [35]. Given these risks, the safety of JC requires careful evaluation. In this study, we confirmed that JC did not inhibit chondrocyte proliferation, supporting its potential for further application.

In vitro, JM and JC exhibited distinct but complementary effects on chondrocyte gene expression. JM enhanced *ACAN* expression and reduced *MMP3* expression under inflammatory conditions, indicating anti-catabolic and cartilage-preserving effects. JC reduced *ADAMTS5* expression in the presence of IL-1β, suggesting a role in limiting matrix degradation. When administered together, JM and JC further increased *ACAN* expression, although this effect was mainly attributable to JM. These results suggest that both JM and JC may serve as promising biomaterials for intra-articular therapy. Importantly, JM also increased *ADAMTS5* and *TNFα* expression under inflammatory conditions, raising concerns that its effects may be context-dependent and potentially detrimental in inflamed joints.

By contrast, the in vivo results were less encouraging. Pain evaluation using the incapacitance test (Figure 5) showed that all MIA-injected groups displayed persistent reductions in weight-bearing compared with non-treated controls. Neither JM alone nor JM combined with HA significantly improved pain-related behavior, unlike HA, which has previously demonstrated symptomatic relief. Macroscopic cartilage assessment (Figure 6) revealed that HA-treated joints exhibited better preservation of both femoral and tibial surfaces compared with JM, and a significant difference was observed between JM and HA + JM in the femoral condyle. Histological evaluation with OARSI scoring (Figure 7) further confirmed that HA and HA + JM, but not JM alone, reduced cartilage degeneration. These findings indicate that JM did not provide consistent structural or symptomatic protection in this short-term MIA model (OAK model), in contrast to HA, which showed reproducible efficacy.

Several factors may explain the discrepancy between in vitro and in vivo findings. JM may be rapidly cleared from the joint cavity, limiting intra-articular exposure. The aggressive and rapid degeneration induced by MIA may overwhelm the protective effects of JM. The use of simple weekly bolus injections may also be suboptimal; advanced delivery methods such as sustained-release microgels or nanoparticle carriers could improve efficacy. Furthermore, JM and JC may be more effective in early or mild OA, where catabolic activity is less severe and reparative pathways remain functional.

One reason we did not obtain similar results to those of Ohta et al. [16] is that the species of jellyfish used as raw material was changed from Nomura’s jellyfish to Moon jellyfish (Aurelia aurita). In addition, we should also consider that the OA model was caused by MIA injection, not traumatic injury.

A key finding from our in vitro experiments may directly explain the disappointing in vivo results. The MIA model is known to create a highly inflammatory intra-articular environment, rich in cytokines like IL-1β. We hypothesize that in such an environment, the detrimental effects we observed with JM—specifically, the upregulation of *ADAMTS5* and *TNFα*—predominated over its beneficial, chondroprotective properties. This potential pro-catabolic shift in a live inflammatory setting could be a primary reason for the lack of a therapeutic effect.

In conclusion, while moon jellyfish-derived JM and JC show promising protective effects at the cellular level, their translation into a therapeutic effect was not observed in this aggressive, inflammatory OA model. Our findings suggest this failure is not only due to methodological limitations such as pharmacokinetics and delivery strategy, but is also potentially rooted in a context-dependent, pro-catabolic effect of JM within a live inflammatory environment. Future studies should therefore focus on two key areas: (1) developing sustained-release formulations to overcome rapid clearance; (2) investigating the signaling pathways responsible for the upregulation of *ADAMTS5* and *TNFα*, to determine whether the therapeutic benefits of JM can be separated from its detrimental effects.

## 4. Materials and Methods

### 4.1. Materials

#### 4.1.1. Isolation of PDs

These experiments used PDs collected from excised excess fingers obtained in polydactyly surgery performed in the Department of Plastic Surgery at our hospital. Chondrocytes from excess fingers were isolated in the same manner as the conventional method performed in our facility [26]. The method is described briefly as follows. The obtained cartilage tissue was finely chopped and placed in an adjusted medium containing Dulbecco’s Modified Eagle’s Medium–nutrient mixture F-12 (DMEM/F12; Gibco, Waltham, MA, USA), with 20% fetal bovine serum (FBS; SAFC Biosciences, Lenexa, KS, USA), 1% antibiotic antimycotic solution (Gibco), to which 5 mg/mL collagenase type 1 (CLS1; Worthington Biochemical Corp., Lakewood, NJ, USA) was added, and the tissue was incubated in an incubator (37 °C, 5% CO_2_, 95% air) for 1.5 h. The tissue suspension was collected through a 100 μm strainer, seeded in a culture plate, and cultured in an incubator under the same conditions. Subsequently, the medium was replaced every 3–4 days and, after confirming that subconfluence had been reached, the cells were cryopreserved after 1 or 2 passages (P1 or P2).

#### 4.1.2. Extraction and Purification of JM

JM was purified by the Jellyfish Research Laboratories (Kanagawa, Japan) using the following method and provided to our hospital. Moon jellyfish, Aurelia sp., was caught in Tokyo Bay or Mikawa Bay, Japan. The water-containing crushed jellyfish tissues were centrifuged at 10,000× *g* at 4 °C for 10 min to remove insoluble materials, and the supernatant was mixed with twice the amount (*w*/*w*) of 2-propanol. The mixture was centrifuged at 10,000× *g* at 4 °C for 10 min, and the pellet was treated with three times the amount (*w*/*w*) of 25% 2-propanol (diluted with pure water). The resultant mixture was centrifuged at 10,000× *g* at 4 °C for 10 min, and the supernatant was mixed with an equal amount of 2-propanol. The pellet was dissolved in water. After centrifugation at 10,000× *g* at 4 °C for 10 min, the supernatant was purified using a 10 kDa cutoff membrane, and the purified fractions were lyophilized. The lyophilized material was dissolved in phosphate buffer and incubated with anion exchange gel beads derivatized with diethylaminoethyl (DEAE) resin (Toyopearl DEAE-650M; Tosoh, Tokyo, Japan) for 1 h. The beads were washed well with phosphate buffer, and the bound proteins were eluted with elution buffer (phosphate buffer plus 0.5 M NaCl). The eluent was collected by filtration, dialyzed against water, and lyophilized.

#### 4.1.3. Extraction and Purification of JC

JC was purified by Jellyfish Research Laboratories using the following method and provided to our hospital. Moon jellyfish, *Aurelia* sp., was caught in Tokyo Bay or Mikawa Bay, Japan. The water-containing crushed jellyfish tissues were centrifuged at 10,000× *g* at 4 °C for 10 min to remove insoluble materials, and the supernatant was mixed with twice the amount (*w*/*w*) of 2-propanol. The mixture was centrifuged at 10,000× *g* at 4 °C for 10 min, and the pellet was treated with three times the amount (*w*/*w*) of 25% 2-propanol (diluted with pure water). The resultant mixture was centrifuged at 10,000× *g* at 4 °C for 10 min, and the pellet was dissolved in water. After centrifugation at 10,000× *g* at 4 °C for 10 min, the supernatant was purified using a 10 kDa cutoff membrane. The purified fractions were lyophilized.

### 4.2. Examination of the Cell Growth-Inhibitory Effects of JM and JC

The cryopreserved PDs (P1) were thawed, diluted with PD medium, and seeded in a CellBIND six-well plate (Corning, NY, USA) at 1.0 × 10^4^ cells/cm^2^. The PD medium comprised DMEM/F12 (Gibco), 20% FBS (*SAFC*), 1% antibiotic antimycotic solution (Gibco), and 0.1% ascorbic acid (Nissin Pharmaceutical, Yamagata, Japan). The medium was replaced 2 days later and, after culturing for another 2 days, upon confirming that it had become subconfluent, the cells were recovered using TrypLE express enzyme (1×) (Gibco). The cells were seeded into two 96-well plates (Corning) with PD medium at a concentration of 3.2 × 10^3^ cells/well. Cell adhesion was confirmed 24 h later, and the medium was replaced with a medium adjusted to achieve JM and JC concentrations of 10%, 3%, 1%, 0.3%, and 0.1% (*n* = 3). One 96-well plate was analyzed 48 h later (24 h after medium replacement) using the CellTiter-Glo 2.0 Assay (Promega). The other plate was analyzed 72 h later (48 h after medium replacement) in the same manner. All culturing was performed in an incubator (37 °C, 5% CO_2_, 95% air). The results were evaluated using a *t* test using SPSS software (v. 26; IBM SPSS Statistics, Armonk, NY, USA).

### 4.3. Cell Culture

For chondrocytes, the cryopreserved PDs (P2) were thawed, diluted with PD medium, and seeded in a CellBIND T150 flask (Corning). Upon confirming that the cells had become subconfluent after 72 h, the medium was discarded and the cells were washed with phosphate-buffered saline and recovered using TrypLE express enzyme (1×) (Gibco). After diluting with PD medium, cells were seeded into a 12-well plate (Corning) at a concentration of 5.0 × 10^4^/cm^2^. Sufficient adhesion of PDs was confirmed 24 h later, and the medium was replaced with medium containing JM 1 mg/mL alone, JC 1 mg/mL alone, and both JM 1 mg/mL and JC 1 mg/mL. IL-1β (20 ng/mL) was added to investigate simultaneously the effect of IL-1β, which is an inflammation-inducing cytokine, on OA (*n* = 4). After the medium was replaced, the cells were cultured for another 24 h, the medium was discarded, and PDs were recovered using TRIzol Reagent (Thermo Fisher Scientific, Tokyo, Japan). All culturing was performed in an incubator (37 °C, 5% CO_2_, 95% air).

### 4.4. RNA Extraction, cDNA Synthesis, and RT-qPCR

RNA was extracted using a RNeasy Mini Kit (Qiagen, Hilden, Germany), and cDNA was synthesized from 1 μg of RNA using a QuantiTect Reverse Transcription Kit (Qiagen). A SimpliAmp Thermal Cycler (Applied Biosystems by Thermo Fisher Scientific, Waltham, MA, USA) performed the reaction at 42 °C for 15 min and then at 95 °C for 3 min. RT-qPCR was performed in accordance with the standard protocols using TaqMan real-time PCR and QuantStudio3 (both from Applied Biosystems by Thermo Fisher Scientific). After reacting at 50 °C for 2 min in a final reaction volume of 20 μL, a total of 40 cycles was performed: 95 °C for 10 min, 95 °C for 15 s, and 60 °C for 1 min. TaqMan Gene Expression Assays (Applied Biosystems by Thermo Fisher Scientific) were used for primers, the details of which are shown in Table 1. GAPDH was selected as the internal control, and each result was evaluated using the 2^−ΔΔCT^ method. The obtained data are listed in the table as the mean ± standard error.

### 4.5. Establishment of MIA Model Rats and Intra-Articular Injections

All procedures using animals in this study were performed in accordance with the Guide for the Care and Use of Laboratory Animals (NIH Publication No. 8023, revised 1978) published by the National Institutes of Health, Bethesda, MD, USA, and the Guidelines of Tokai University on Animal Use. This animal experiment was approved by the Animal Committee of Tokai University (Ethics Approval Number 201063).

#### 4.5.1. Animals

A total of 30 male Wistar rats were purchased at 6 weeks of age (CLEA Japan Inc., Tokyo, Japan) and acclimated for 2 weeks before the start of the experiments. All experiments were initiated when the rats were 8 weeks old. The animals were housed under normal conditions for 2 weeks before the start of the experiments to acclimate them to the environment. One to two rats were housed per cage in sterile conditions, and rodent chow and water were allowed ad libitum.

#### 4.5.2. Induction of OA by MIA

**Figure 8:**
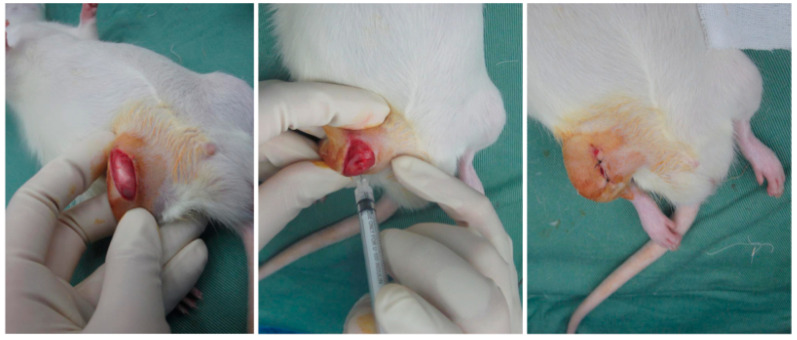
Procedure of Intra-articular Injection in Rats. Under inhalation anesthesia, an incision is made in the skin of the right knee to expose the joint capsule. The injection is then administered through the patellar tendon. The same procedure was used for all injections, including the MIA injection and the subsequent treatments.

We used 30 male Wistar rats at 8 weeks of age. A total of 30 rats were randomly assigned to five groups (*n* = 6 per group). MIA injections were administered to four of these groups. With the animal under anesthesia with isoflurane and oxygen inhalation, right knees were disinfected. Under sterile conditions, a 1 cm incision was made in the skin via the medial parapatellar approach to expose the patellar tendon. The knee joint was flexed and MIA (0.2 mg dissolved in 40 mL of physiological saline; Sigma-Aldrich, St. Louis, MO, USA) was injected into the knee joint through a 27 G needle. After injection, the skin was sutured with nylon sutures (Figure 8).

#### 4.5.3. Intra-Articular Injection

**Figure 9:**
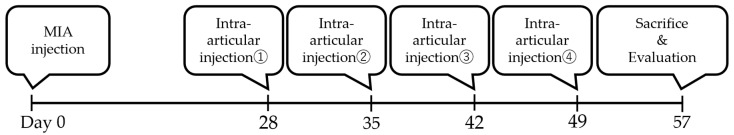
Schedule for MIA injection, intra-articular injection, and sacrifice. The injected solutions consisted of either saline, JM, HA, or JM and HA.

Figure 9 presents the experimental schedule. On Day 0, arthritis was induced in all rats via an intra-articular injection of MIA. Starting from day 28, all 4 treatment groups received intra-articular injections (40 μL) once a week, for a total of 4 injections (on Days 28, 35, 42, and 49). The volume of all intra-articular injections was 40 µL. The injected substances were as follows: saline (control group), JM (400 µg/40 µL), hyaluronic acid (400 µg/40 µL) (HA; Artz^®^, Kaken Pharmaceutical, Tokyo, Japan), and a mixture of JM (200 µg/20 µL) and HA (200 µg/20 µL). The fifth group served as an untreated control.

#### 4.5.4. Pain Evaluation

An incapacitance meter (BrainScience Idea Co., Ltd., Osaka, Japan) was used to detect changes in the distribution ratio of the damaged limb to the undamaged limb, and the ratio served as the gauge for evaluating pain. This device is used widely to investigate pain and pain-alleviating effects [27,28,29,30]. The measurements were made when the rat’s hind legs were both positioned over the platforms and the rat was stationary. The weight distribution of both hind legs was measured 10 times, and the following formula was used to calculate the limb weight distribution ratio (%). (Damaged limb load (g)/(undamaged limb load (g) + damaged limb load (g)) × 100).

After MIA injection, the weight distribution was measured 14 times (Days 1, 3, 7, 14, 21, 27, 31, 35, 38, 41, 45, 49, 52, and 56).

#### 4.5.5. Evaluation

The rats were sacrificed by an overdose of 50% isoflurane. The knee joint was exposed using the medial parapatellar approach and the femur and the tibia were separated. Each articular surface was stained using indigocarmine (Alfresa Pharma Corporation, Osaka, Japan). Evaluation was performed using macroscopic cartilage and bone scores, referring to the method of Udo et al. [24]. Scoring was performed by three single-blinded examiners.

The femur and tibia were fixed in 20% formalin (Wako Pure Chemical, Osaka, Japan) for 6 days. Each section was degreased and demineralized with acetone solution (Wako Pure Chemical) and ethylenediaminetetraacetic acid (EDTA) solution (Wako Pure Chemical). They were fixed again with 20% formalin, decalcified with EDTA solution, and embedded in paraffin wax. The section of the medial femoral condyle was cut in the sagittal direction (3 μm sections) and they was stained with Safranin O in a 0.08% fast green aqueous solution and 0.1% Safranin O aqueous solution. Microscopic images were captured under a BZ-9000 Biorevo fluorescence microscope (Keyence, Osaka, Japan). We evaluated the distal part of the femoral articular cartilage and the intermediate region of the articular cartilage of the tibia based on the cartilage region determination method of Nomura et al. [31]. Evaluation was performed OARSI scores [25], and scoring was performed by three single-blinded examiners.

### 4.6. Statistical Analysis

One-way analysis of variance was performed using SPSS software (v. 26; IBM SPSS Statistics). Significant differences were identified by Tukey’s method and the Games–Howell method.

## 5. Conclusions

In vitro, JM and JC suppressed catabolic gene expression and promoted ACAN expression, suggesting cartilage-protective effects. In vivo, however, intra-articular JM, alone or with HA, did not improve pain, cartilage integrity, or histology in the MIA-induced OA rat model.

## Figures and Tables

**Table 1:** List of primers used in RT-qPCR.

Primer	ID
COL1A1	Hs00164004_m1
COL2A1	Hs00264051_m1
SOX9	Hs01001343_g1
MMP3	Hs00968305_m1
MMP13	Hs00233992_m1
ACAN	Hs00153936_m1
TGFβ	Hs00998133_m1
TNFα	Hs00174128_m1
ADAMTS5	Hs00199841_m1
GAPDH	Hs02758991_g1

Catalog number is #4331182.

## Data Availability

The data presented in this study are available upon reasonable request from the corresponding author. The data are not publicly available due to privacy.

## Abbreviations

AA	Ascorbic acid
AB	Antibiotic antimycotic solution
ACAN	Aggrecan
ADAMTS5	A Disintegrin and Metalloproteinase with Thrombospondin motifs 5
ASC	Adipose derived stem cell
COL1	Type I collagen
COL2	Type II collagen
Ct	Cycle threshold
DMEM/F12	Dulbecco’s Modified Eagle’s Medium–nutrient mixture F-12
FBS	Fetal bovine serum
GAPDH	Glyceraldehyde-3-phosphate Dehydrogenase
HA	Hyaluronic acid
IL-1β	Interleukin-1β
JC	Jellyfish collagen
JM	Jellyfish mucin
MMP3	Matrix Metalloproteinase-3
MMP13	Matrix Metalloproteinase-13
MUC5AC	Mucin 5ac
OA	Osteoarthritis
PBS	Phosphate-buffered saline
PRP	Platelet-rich plasma
RT-qPCR	Quantitative reverse-transcription polymerase chain reaction
TGFβ	Transforming growth factor-β
TNFα	Tumor necrosis factor-α
